# Leadership Style and Hospital Performance: Empirical Evidence From Indonesia

**DOI:** 10.3389/fpsyg.2022.911640

**Published:** 2022-05-26

**Authors:** Mochammad Fahlevi, Mohammed Aljuaid, Sebastian Saniuk

**Affiliations:** ^1^Management Department, BINUS Online Learning, Bina Nusantara University, West Jakarta, Indonesia; ^2^Department of Health Administration, College of Business Administration, King Saud University, Riyadh, Saudi Arabia; ^3^Department of Engineering Management and Logistic Systems, Faculty of Economics and Management, University of Zielona Góra, Zielona Góra, Poland

**Keywords:** leadership style, hospital performance, organizational identification, private hospital, Indonesia

## Abstract

The COVID-19 pandemic created new conditions for the functioning of all organizations. Suddenly, there was a problem with the lack of appropriate leadership styles models in health care organizations (hospitals), which are particularly vulnerable to disruptions in a pandemic. Hospitals, in particular, have become exposed to organizational and managerial problems. The article aims to propose an appropriate leadership style model that will guarantee a high level of hospital efficiency, taking into account a pandemic situation in the example of private hospitals in Indonesia. Organizational identification is promoted as a mediating variable due to the high level of this variable in explaining hospital performance in Indonesia based on preliminary studies. During research used a structural equation model using 394 samples at the unit leadership level in private hospitals in Indonesia. The results of this study explain that there is an impact between innovative leadership and strategic leadership styles on hospital performance. Private hospitals in Indonesia need to improve themselves to use the most appropriate leadership style model based on the needs of the hospital itself.

## Introduction

The COVID-19 pandemic began in 2020 and has significantly impacted all socio-economic aspects, including industry, services, education, and health (Kaye et al., 2021). Almost all areas faced difficulties. Particular attention should be paid to health care, which requires a lot of innovation and development to help fight the effects of the COVID-19 pandemic. Hospitals are one of the fundamental pillars of coping with the consequences of the COVID-19 pandemic (Algaissi et al., 2020). In addition to public hospitals dedicated to rescuing COVID-19 patients, other hospitals are needed to accommodate other patients. The COVID-19 phenomenon has significantly impacted the performance of private hospitals in Indonesia, especially those listed on the Indonesian Stock Exchange (Fahlevi, 2021). Private hospitals can support the treatment of both COVID-19 patients and organize the treatment of other diseases. The versatility and flexibility of private hospitals can help provide adequate and standardized health services to the public. An analysis of hospitals’ financial statements will assess their activities during the COVID-19 pandemic. It is known that there has been a decline in the performance of hospital companies on the Indonesian stock exchange.

Hospital services during the COVID-19 pandemic experienced a significant decline in the 2nd quarter of 2020 (Fitra, 2020). At that time, COVID-19 had not been comprehensively understood, resulting in a decline in services due to anticipation from the public delaying visiting the hospital, or it could also be from the precautionary factor of the hospital, which limits services so that the volume of services decreases. In addition, from the hospital side, there is a possibility that it is not a COVID-19 referral hospital. Good service to customers remains the hospital’s main goal so that customers do not make a bad assessment of the hospital’s performance as a health service (Dinata et al., 2019). The hospital as a health service organization consists of parts that are interdependent on each other. Every organization requires coordination and communication so that each part of the organization works according to its responsibilities. Without coordination, it is difficult for hospitals to function properly (Sunarsi et al., 2020; Supriyadi et al., 2020).

The mission of hospitals is to treat patients using the latest achievements in medicine while providing a high level of quality medical services (Bambra et al., 2008). This means the need for continuous improvement of the organization management and improvement of the competencies of employed doctors and staff. This approach represents a competence maximization process that encourages high-performance culture (Fahlevi and Alharbi, 2021). Leadership within an organization also significantly impact achieving good organizational performance. To achieve the highest level of employee development, human resource management, harmonious working relationships between employees and leadership styles are expected to increase labor productivity (Kadiyono et al., 2020). Effective leadership can provide direction to the efforts of each employee in achieving organizational goals, and in managing his subordinates, a leader must be able to read the right situation so that he can provide views on dealing with and solving problems (Al-Sada et al., 2017). Good leadership is needed to develop employees and organizational identification to increase productivity. The phenomenon found by researchers in the pre-study shows that leadership style and identification organization have an influence on employee performance in private hospitals in Indonesia (Arab et al., 2007; Risambessy et al., 2012; Yunarsih et al., 2020). From the data obtained from private hospitals, there are indications that in 2020 there was a pandemic explosion. Several hospital performances have decreased due to the unpreparedness of private hospitals to face a drastic increase in the number of patients during the pandemic (Alharbi et al., 2022).

Several hospital performances have decreased due to the unpreparedness of private hospitals to face a drastic increase in the number of patients during the pandemic (Alharbi et al., 2022). In 2020 there was a change in the management ranks in several private hospitals. At this time, the performance of hospitals and health workers is also significant to look into to keep and improve people’s health. This phenomenon is a question mark for researchers as to whether there is an influence between leadership style and organizational identification on hospital performance.

Over the last three decades, healthcare organizations (particularly hospitals) have increasingly embraced governance models that are not based on the conventional “professional bureaucracy” (Alwashmi, 2020), and are inspired by managerialist philosophy. In the public sector, these changes have resulted in the “corporatization” of hospitals toward a more business-like structure, with the goal of increasing decision-making independence. This transformation necessitated significant structural changes in leadership positions, including the establishment of the Chief Executive Officer (CEO) position and the appointment of boards of directors (Aras and Crowther, 2008). Following the fundamental premise of corporate governance that improved governance mechanisms result in increased organizational efficiency and effectiveness, the adoption of these business-like governance arrangements has been viewed as critical for performance improvement in private (for-profit and not-for-profit) and public hospitals. While these reforms have garnered general approval in terms of improved organizational autonomy and increased independence in strategic decision-making processes, the question of what combination of experience and skills hospital senior leaders should possess remains unanswered (Johansen and Sowa, 2019).

In this study also used the mediator variable organizational identification which is believed to have a correlation with the existence of transformational leadership, innovative leadership, and strategic leadership style. Many approaches to leadership research have suggested that the concept of organizational identification can act as a mediator of leadership effectiveness (Van Knippenberg et al., 2004). Organizational identification reflects the degree of overlap between self-identity and organizational identity (Van Knippenberg and Sleebos, 2006). The more people identify with an organization, the more values, goals, and organizational norms are embedded in the individual’s self-concept. The results of previous research suggest that when employees already have a high identification with their organization, the effectiveness of leader behavior in increasing the ability of employees to adapt will work better. Employees are willing to adapt to fit the organizational system when they see themselves as members of the organization (Carmeli et al., 2007). Conversely, if employees are not identified with the organization that employs them, it is difficult for them to find meaning in the workplace (Ashforth et al., 2008). In this situation, transformational leadership, innovative leadership, and strategic leadership styles are more needed and should have a stronger effect.

In this regard, scholars and practitioners have questioned whether the hospital’s chief executive officer and board of directors should be clinically trained top executives (Kakemam and Goodall, 2019; Davlyatov et al., 2021; Moores et al., 2021). The empirical evidence suggests that clinicians’ involvement in leadership positions varies significantly across countries. Understanding the leadership style used to manage the hospital is critical at this point. The leader’s decisions will determine the organization’s fate. As individuals who are followed by their subordinates, leaders must be capable of motivating and directing their subordinates to accomplish organizational goals (Fahlevi et al., 2019).

## Literature Review

Leadership has probably received the most written about, formal research, and informal discussion of any other major topic. Despite this increased focus on leadership, there is still debate. For instance, Bennis (2018) emphasizes in one of his articles titled “The End of Leadership” that effective leadership is impossible without employees’ full involvement, initiative, and cooperation. In other words, a great leader cannot be a great follower (Colls-Senaha, 2022). Posner (2021) makes the following observations about the necessary changes in Bisoux’s (2002) view of business leadership: “Historically, businesspeople believed that a leader should be like a ship’s captain: cool and collected. Now we see that leaders must possess human characteristics. They must be able to relate, be empathic, and be around people. Leaders must be a part of the action, not aloof from it.” Today’s organizations need influential leaders who understand the challenges of the turbulent environment. Uncertain conditions require the leader to have a good relationship with the employees to increase their employees’ effectiveness (Nanjundeswaraswamy and Swamy, 2014).

An interesting issue is a leadership in a crisis, which requires the formulation of the challenge ahead, full recognition of the contributions so far and an optimistic grasp of the reality that we will eventually emerge from the crisis and return to normal (Wibowo et al., 2020). According to the many researchers, in addition to the need for research related to a better understanding of the virus, its epidemiology and strategies for preventing and treating COVID-19 disease, there is also a clear need to answer the question of how to lead in healthcare in crisis situations and the need to catalog best leadership practices (Stoller, 2020). Shingler-Nace identifies five elements to successful leadership during this crisis: Staying calm, communication, collaboration, coordination, and providing support (Shingler-Nace, 2020). The readiness of health workers is the main discussion during a pandemic (Chirico et al., 2020), as experienced by every country when the pandemic reaches its peak, especially in Indonesia, which has centralized health services only in big cities (Fahlevi, 2020). The lack of tracing of the population infected with the virus is also a problem for health workers in serving the overwhelming number of patients, so this incident causes health workers to have a high risk of being infected with the virus, especially in hospitals (Rees et al., 2020).

COVID-19 raises many ethical medical dilemmas for leaders of healthcare institutions. Treatment rationale is one of the major problems faced by healthcare professionals (Shryock and Meeks, 2020). As the number of cases increases, it is unclear how leaders should fairly rationalize scarce resources. During the crisis, leadership is of utmost importance in helping to solve these medical dilemmas. A recognized characteristic of decisive leadership is rapid response, based on a clear understanding of the threat posed by COVID-19 and that delayed action could lead to worse outcomes.

Tay et al. (2021) indicated the following assumptions in the management of the health care institutions (hospitals):

– in a pandemic, regular, and directed meetings by a smaller leadership core group are required, for prompt decision making and execution of action plans;
– the military format, with domain groups to look at manpower, intelligence, operations, and logistics matters, is useful in managing a pandemic;
– discipline, flexibility, and teamwork with strong focus on infection prevention and control, and patient and staff safety as well as staff wellbeing are key principles for leadership teams managing a pandemic.

Leadership or leadership is the most basic thing in managing an organization. The absence of leadership or leadership will make organizations such as hospitals and health facilities lose direction throughout their service journey. In addition, there will also be a lack of innovation, creativity, independence, responsibility, and a series of other strategic attitudes. If continued, it will jeopardize the stability and sustainability of hospitals and health facilities in carrying out the mandate of the law to provide complete health services to the community. Therefore, leadership is a very important pillar in the management of hospitals and health facilities. Hospitals and health facilities have business characteristics that are labor-intensive, capital-intensive, technology-intensive to problem-intensive (Al-Hashimi and Al-Hashimi, 2020). Leadership policies can determine the direction of hospital adjustment in pandemic conditions that are very different from normal conditions. Government policies in dealing with the surge in patients are difficult to implement in every hospital that has a fairly high gap, especially regarding isolation facilities and breathing apparatus. Hospital readiness in facing a pandemic can be said to be a complex problem due to many factors that can determine why not all hospitals can handle all patients. In the case of Indonesia, there are many cases where some hospitals refuse patients because of the unavailability of rooms at their hospitals (Manusubroto et al., 2020).

**FIGURE 1:**
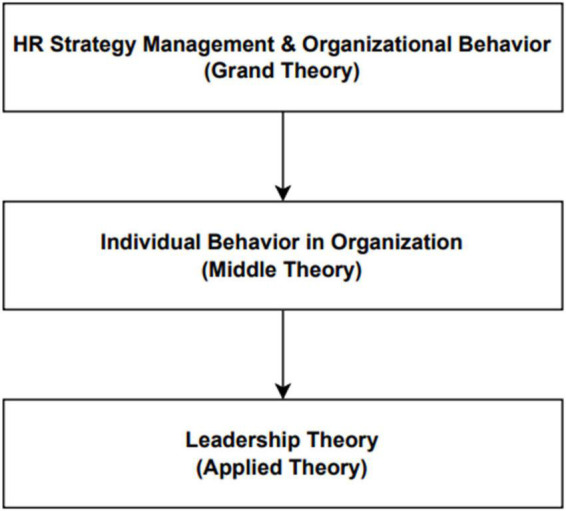
Theory of leadership.

As illustrated in Figure 1, leadership theory evolved from the grand theory of human resource management strategy and organizational behavior, which was extensively discussed by Robbins and Luthans at the outset and further developed as the theory of individual behavior in organizations, which discusses the role of individuals in determining an organization’s goals and accomplishments. Individuals are viewed as critical in either achieving the company’s objectives or diminishing the company’s value based on the performance and decisions made by each individual organization. People will be able to break down a lot of different organizational structures into employees and leaders, which will make it easier to talk about the leadership theory at the heart of this research, which is about how a CEO leads a private hospital in Indonesia.

Although research on leadership can be traced back to Plato’s ideas in the West and Sun Tzu’s ideas in the East, it appears that no single definition has come close to consensus on its fundamental meaning. It should be obvious that we do not require additional “lists” of leadership competencies or skills, as leadership research appears to be a method for determining “the truth” about leadership. The more time we devote to studying leadership, the more complicated the concepts become (Doh and Stumpf, 2005).

### Hypothesis Development

Zuraik and Kelly (2018) have investigated the relationship between CEO transformational leadership, innovation climate, and firm performance. The results of Abdelrahman and Louise’s research show that CEO transformational leadership has a positive direct relationship with company performance and has an indirect effect on company performance. Carreiro and Oliveira (2019) found a positive effect of transformation leadership on company performance. The explanation above provides information that the leadership factor as described has a strong influence on firm performance, so in this study, the leadership variable is used as the basic variable to measure its effect on hospital performance. Leadership, in theory, is an important factor in making decisions and determining the direction of the hospital strategy going forward. The position of the highest leadership in the hospital, which is often associated with organizational identification (Carmeli et al., 2007), provides its own climate regarding leadership in hospitals, which is still rarely studied, especially regarding leadership factors in depth. The application of transformational leadership is not that easy in a fairly complex company, such as a hospital.

*Hypothesis 1: Transformational leadership has a significant effect on hospital performance*

*Hypothesis 2: Transformational leadership has a significant effect on Organizational Identification*

Somsueb et al. (2019) found a positive influence of innovative leadership on firm performance, but this research is only in the world of education or companies working in education in business-based companies. Research on innovative leadership is still new and is still a psychological study. Innovative Leadership is a new variable construction and no researcher has used this variable yet. Khalili (2016), the founder of Innovative Leadership, produced significant and positive findings on creative and innovative behavior at the individual level in the workplace. As a result, innovation in leading companies is an important factor in hospitals because they have to compete in a perfectly competitive market so that the factors that drive companies toward leading the market will continue to be expected by the government and as a common goal of creating wealth and social impact for society. Hospital management is required to create an organizational identification in each of its companies (Zhang and Chen, 2013), namely by creating continuous innovation in organization. As previously described in previous research, innovative leadership factors are assumed to have a very strong influence on hospital performance and organizational identification.

*Hypothesis 3: Innovative leadership has a significant effect on hospital performance*

*Hypothesis 4: Innovative leadership has a significant effect on Organizational Identification*

According to Satyawati and Suartana (2014) has conducted research on the influence of strategic leadership style and organizational culture on job satisfaction, which has an impact on firm performance. The results of this study indicate that leadership style has a positive effect on job satisfaction and firm performance. Elements of transactional leadership, such as providing conditional rewards, have been found to be positively related to employee performance and effort (Bass, 1990). However, when a situation occurs where the company faces a complicated and uncertain situation, the leadership spirit will face challenges and tend to decline. Interestingly, recent research has also found a positive relationship between transactional leadership and diversity practices when leaders are older and have higher social values (Ng and Sears, 2012). Because of the limitations of transactional leadership, it often contrasts (unfavorably) with transformational (Tucker and Russell, 2004; Vera and Crossan, 2004). Not surprisingly, many empirical studies show that transformational leadership has greater performance and results. For example, at lower organizational levels, transactional leadership has been found to have a positive relationship with the mean outcome of organizational identification, but the relationship is not as strong as with transformational leadership (Epitropaki and Martin, 2005). In much of the literature on strategic leadership styles, in the context of strategic leadership, transformational leadership, transactional and paternalistic styles have an effect on firm performance. The effects are thought to vary in intensity. It is assumed that a transformational leadership style has the greatest impact on organizational performance (Özer and Tınaztepe, 2014).

*Hypothesis 5: Strategic leadership style has a significant effect on hospital performance*

According to Widianto and Harsanto (2017) found the positive influence of transformational leadership on firm performance, both directly and indirectly. This research was conducted in Indonesia and can be used as a reference for further studies in this study, but this research does not specialize in hospitals as the purpose of this study. Leadership can be distracted by several factors that make the effectiveness of leaders not reach the organization as a whole, so this study tries to answer leadership factors directly and indirectly, which can measure the magnitude of influence and mediate other variables in determining hospital performance. Organizational identification, in turn, will positively predict hospital performance for two reasons. First, employees who have a strong identification with their organization have positive attitudes toward themselves. Researchers state that the perception of organizational unity appears as part of increasing self-esteem (Tajfel, 1978; Hogg and Turner, 1985). In this sense, higher levels of self-esteem can result in greater employee effort (Walumbwa et al., 2008). Identification also motivates employees to act in support of the interests of the organization. As a result, this greater effort and motivation helps employees focus and be more effective at their tasks and improve their individual performance. Previous research has shown that organizational identification is associated with outcomes such as role behavior and firm performance (Riketta and Van Dick, 2005; Walumbwa et al., 2008, 2011). Second, individuals who perceive themselves as part of an organization see collective interests as self-interest, which motivates behavior to support the collective (Van Knippenberg and Sleebos, 2006). As noted by Van Knippenberg and Sleebos (2006), employees who identify more with their organization are more likely to engage in behaviors that go beyond their basic roles. Recently, Xu et al. (2019) found a positive relationship between organizational identification and firm performance. Based on the arguments above, the identification of organizations that mediate the relationship between transformational leadership and hospital performance.

*Hypothesis 6: Transformational leadership has a significant effect on hospital performance mediated by organizational identification*

Organizational identification can act as a mediator of leadership effectiveness (Van Knippenberg et al., 2004). Organizational identification reflects the degree of overlap between self-identity and organizational identity (Van Knippenberg and Sleebos, 2006). The more people who identify with an organization, the more values, goals, and organizational norms are embedded in the individual’s self-concept. The results of previous studies suggest that when employees already have a high identification with the organization, the effectiveness of leader behavior in improving employee adaptability can be successful. Employees are willing to adapt to fit into the organizational system when they see themselves as members of the organization (Carmeli et al., 2007). Moreover, they are basically motivated to behave according to the goals and norms of the organization, so the subordinates have a low need for leadership. This study proposes organizational identification as a mediator between the variables of innovative leadership and hospital performance.

*Hypothesis 7: Innovative leadership has a significant effect on hospital performance mediated by organizational identification*

**FIGURE 2:**
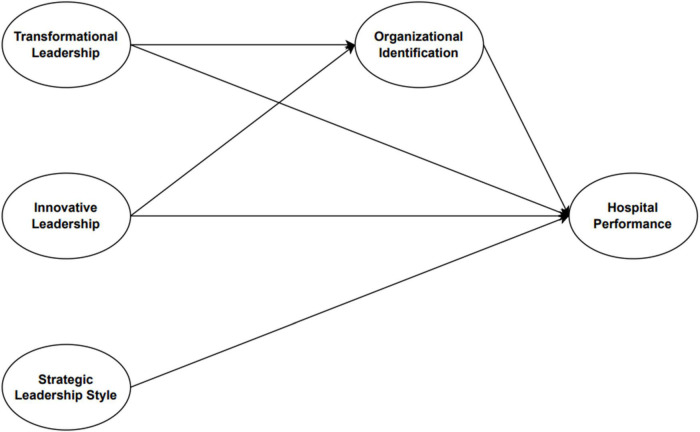
Research model.

Based on the explanation above, it can be formulated a new research model that has variable novelty compared to previous research that organizational identification has a mediating role between leadership and hospital performance in the model proposed in Figure 2.

## Materials and Methods

The population for this study was sampled using a stratified cluster random sampling technique, as the population is divided into two levels: one level and two levels below the CEO of a private hospital. The sample is drawn from the population to be studied in this section. For sampling purposes in this study, the sample was divided into two groups: the primary source sample and the instrument trial sample. The test sample is charged with determining whether the instrument is valid and reliable enough to be used in the study, and 30 individuals will be assigned to test the instrument. The trial was conducted prior to the primary research. According to Hair et al. (2011), a SEM model with up to five latent variables (constructs) and each construct described by three or more indicators is considered adequate when the sample size is between 100 and 150. To meet the sample size requirements for SEM analysis, the sample size should be between 100 and 200, and at least five times the number of indicators. This study involved 394 participants who filled out the questionnaire in person or online.

The variables in this study are classified as latent variables (unobserved variables) and manifest variables (observed variables), which are formed by two structures. There are three exogenous latent variables in the substructure, namely the independent variable that influences the dependent variable, CEO transformational leadership, leadership innovation, and strategic leadership style, as well as the mediating variable, organizational identification. The endogenous latent variable is hospital performance. The variables in Table 1 were created to quantify the determination, which serves as a theory for quantifying these variables. Indicators are derived from dimensions, which are the conceptual variables’ derivations. To provide context for the questionnaire and to simplify it, an operational definition of the variable is created as a derivative of the conceptual variable. Each variable was quantified operationally using a Likert scale ranging from 1 to 5, with the following interpretations: strongly disagree score 1, strongly disagree score 2, neutral score 3, agree score 4, and strongly agree score 5.

**TABLE 1:** Measurement.

No	Indicators	Item	Sources	Scale
**Transformation leadership**				
1	*Vision*	• CEO emphasizes the use of intelligence to overcome obstacles/obstacles • CEO can read market conditions	Carreiro and Oliveira, 2019	Likert scale 1–5
2	*Intellectual stimulation*	• I take pride in communicating and associating with my CEO • CEO prioritizes knowledge over expertise		
3	*Inspirational communication*	• CEO is always motivating • I learned a lot from my CEO		
4	*Supportive leadership*	• CEO gives me reasons to change the way the problems I think about • My CEO can identify company risks		
5	*Personal recognition*	• CEO tries to build an emotional connection with employees • CEO is different from the previous boss		
***Innovative leadership***				
1	*New idea*	• CEO has a brilliant idea • CEO has foresight for the company	Zacher and Rosing, 2015	Likert scale 1–5
2	*Implementation*	• CEO always tries to make all programs work • CEO always provides oversight to the obstacles of the planned program		
3	*Improvement*	• CEO makes important changes to the company • CEO has a new innovation in managing the company		
4	*Process and routine*	• CEO creates a new culture within the organization • CEO is very detailed in every company’s performance		
***Strategic leadership style***				
1	*Transformation*	• CEO always sets a good example for employees • Encourage employees to develop competencies	Özer and Tınaztepe, 2014	Likert scale 1–5
2	*Transactional*	• CEO always gives appreciation for achievements • CEO is very firm on violations		
3	*Paternalistic*	• CEO treats us fairly • CEO is very tolerant of differences		
***Organizational identification***				
1	*Personal*	• CEO fulfills the rights and obligations of every individual in the company • CEO always asks for our opinion	Wang et al., 2017	Likert scale 1–5
2	*Employee*	• CEO fosters employee’s sense of responsibility toward the company • CEO applies work-life balance for employees		
3	*Organization*	• CEO synergizes work with company goals • CEO puts company interests ahead of personal interests		
***Hospital performance***				
1	*Operational excellence*	• CEO fixes every problem in the company • Company management is getting neater under my CEO	Wu and Chen, 2014	Likert scale 1–5
2	*Customer intimacy*	• CEO maintains the good name of the company • CEO maintains the company’s relationship with stakeholders		
3	*Product leadership*	• CEO makes regular improvements to the company’s performance • CEO is able to bring the company to compete with other companies		
4	*Financial achievement*	• CEO makes the company big profits • CEO is able to control the company’s finances for the better		

In this study, it is known that the research model has many variables and consists of many indicator items so that the use of bootstrapping with SmartPLS 3 software is the right choice because the research sample is in the medium category. Bootstrapping method is more often used in structural equation models. The SmartPLS program only provides one resampling method, namely bootstrapping which consists of three schemes, namely the no sign changes scheme, individual sign changes and construct level changes schemes. The scheme suggested by smartPLS (default) is construct level changes because this scheme provides loose assumptions so that T-statistics increases because it only uses a loading score measure of a direct relationship between latent variables and indicators. Bootstrapping was chosen because to assess the level of significance or probability of direct effects, indirect effects and total effects. In addition, bootstrapping can also assess the level of significance of other values, including: r square and adjusted r square, f square, outer loading, and outer weight.

## Results of Research

The Average Variance Extracted (AVE), Outer Loading, Cronbach’s Alpha, and Composite Reliability are used to measure the indicator (outer model). As indicated by the outer loading of each variable indicator, outer validity is used to determine the indicator’s validity as a measure of the variable. If the outer loading value for each indicator is greater than 0.70, the indicator is said to be reliable (in research in undeveloped fields, it can be 0.5–0.6) (Hair et al., 2017). If the standard value of outer loading is greater than 0.70, then all loading values of less than 0.70 are removed from the model (Ringle et al., 2020). The Average Variance Extracted-AVE method is used to determine whether the discriminant validity requirements have been met. A total of 0.50 is the minimum value required to declare that validity has been achieved. Construct Reliability attempts to measure the construct’s reliability. The value that is considered reliable must be greater than 0.70. Cronbach’s alpha and composite reliability are associated with construct reliability (Lind et al., 2018).

**FIGURE 3:**
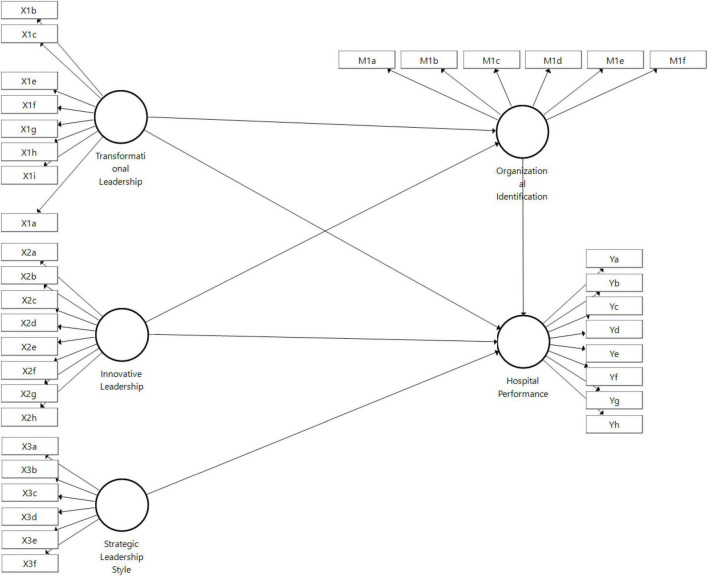
Outer model.

As shown in Table 2, two items from the variable transformational leadership have a value of less than 0.7, namely X1d and X1j, implying that the latter will be removed from the model that already exists due to a failure to meet the model’s outer loading standard. Once the second item is completed, the model’s illustration will revert to the one represented in Figure 3.

**TABLE 2:** Validity and reliability.

Construct	AVE	Outer loading	Cronbach’s alpha	Composite reliability
Transformational leadership	0.608		0.925	0.938
• X1a		0.706		
• X1b		0.804		
• X1c		0.853		
• X1d		0.607		
• X1e		0.838		
• X1f		0.869		
• X1g		0.858		
• X1h		0.836		
• X1i		0.784		
• X1j		0.505		
Innovative leadership	0.784		0.960	0.967
• X2a		0.883		
• X2b		0.911		
• X2c		0.878		
• X2d		0.906		
• X2e		0.902		
• X2f		0.920		
• X2g		0.848		
• X2h		0.832		
Strategic leadership style	0.780		0.943	0.955
• X3a		0.889		
• X3b		0.902		
• X3c		0.908		
• X3d		0.833		
• X3e		0.894		
• X3f		0.869		
Organizational identification	0.790		0.947	0.958
• M1a		0.895		
• M1b		0.856		
• M1c		0.920		
• M1d		0.858		
• M1e		0.925		
• M1f		0.877		
Hospital performance	0.788		0.961	0.967
• Ya		0.894		
• Yb		0.868		
• Yc		0.867		
• Yd		0.860		
• Ye		0.915		
• Yf		0.910		
• Yg		0.885		
• Yh		0.900		

In the next stage, a discriminant validity test will be carried out, which aims to test to what extent the latent construct is really different from other constructs. A high discriminant validity value gives an indication that a construct is unique and able to explain the phenomenon being measured. This value is a cross loading factor value that is useful for determining whether a construct has adequate discriminant by comparing the loading value on the intended construct, which must be greater than the value of loading with another construct (Hair et al., 2017).

From Table 3 above, it can be seen that all loading indicators on the construct are > cross-loaded. For example, in the hospital performance construct, where the loading value of all indicators is greater than all cross loadings to other constructs, the value of loading for one construct is greater than the cross-loading for another construct. Discriminant validity has been met because all indicators of the loading value of the construct are greater than cross loading. The next stage is hypothesis testing with bootstrapping, which is a process to assess the level of significance or probability of direct effects, indirect effects, and total effects. In addition, bootstrapping can also assess the level of significance of other values, including r square. In the complete PLS SEM bootstrapping method, all values that can be analyzed in the partial least squares analysis are bootstrapped to produce the probability value (Hair et al., 2017).

**TABLE 3:** Fornell-Larcker criterion.

Construct	Hospital performance	Organizational identification	Transformational leadership	Innovative leadership	Strategic leadership Style
Hospital performance	0.915				
Organizational identification	0.888	0.901			
Transformational leadership	0.877	0.863	0.908		
Innovative leadership	0.899	0.885	0.889	0.887	
Strategic leadership Style	0.894	0.878	0.880	0.883	0.832

**FIGURE 4:**
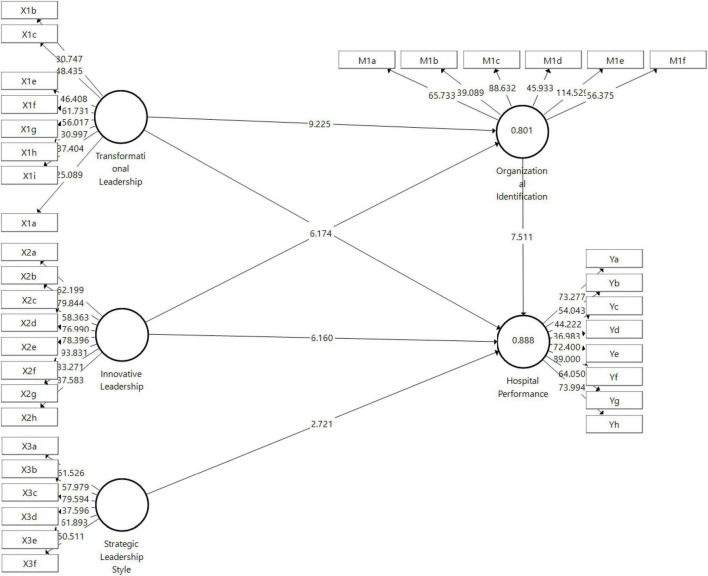
Path analysis.

Based on Figure 4, the path coefficient values range from −1 to + 1. The closer the value is to + 1, the stronger the relationship between the two constructs. The relationship that is closer to -1 indicates that the relationship is negative (Sarstedt et al., 2017). Based on the table in the image of bootstrapping direct effects above, it can be thought of as the following:

In Table 4, it is shown that all hypotheses in this study were accepted except for hypothesis 1, which was rejected because the *P*-value was greater than 0.05, meaning that the transformational leadership hypothesis on hospital performance was rejected. It is also known that the R-square value of the two endogenous variables, namely good enough organizational identification, has an R-square of 0.801 or 80.1% and hospital performance of 0.888 or 88.8%, so that this research model can be said to be very good in explaining hospital performance.

**TABLE 4:** Path coefficients.

Hypothesis	Path	Original sample	Sample mean	STDEV	*T*-statistics	*P*-values	Supported
Hypothesis 1	Transformational leadership → Hospital performance	0.035	0.041	0.053	0.662	0.508	No
Hypothesis 2	Transformational leadership → Organizational identification	0.547	0.546	0.059	9.225	0.000	Yes
Hypothesis 3	Innovative leadership → Hospital performance	0.351	0.353	0.057	6.160	0.000	Yes
Hypothesis 4	Innovative leadership → Organizational identification	0.370	0.371	0.060	6.174	0.000	Yes
Hypothesis 5	Strategic leadership style → Hospital performance	0.154	0.151	0.057	2.721	0.007	Yes
Hypothesis 6	Transformational leadership → Organizational identification → Hospital performance	0.241	0.238	0.042	5.774	0.000	Yes
Hypothesis 7	Innovative leadership → Organizational identification → Hospital performance	0.163	0.162	0.034	4.814	0.000	Yes

## Discussion

In discussing the dimensions of the CEO transformational leadership variable, it can be seen that the strongest dimension is the supportive leadership dimension. This provides information that supports the leadership is the most effective thing to do in private hospitals to maximize the transformation carried out by hospital leaders. Transformational leadership is often contrasted with transactional leadership (Avolio and Bass, 2002), which focuses on promoting the interests of individual leaders and their followers. Transformational leaders empower followers and pay attention to their individual needs and personal development, helping them develop their own leadership potential through coaching, mentoring, and support, and challenging them to solve problems innovatively. This is particularly important in crises, such as the COVID-19 pandemic, where the leader is forced to act in a non-standard manner, to be open to innovative solutions. Supportive leadership is a leadership style in which the CEO not only delegates tasks and receives results, but instead supports employees until the task is completed. The main plus side of supportive leadership is that the CEO will work with the employee until he or she is empowered and skilled enough to handle tasks with minimal supervision in the future. One aspect that emphasizes supportive leadership is teamwork. The CEO begins by creating a team of skilled individuals who can perform the task at hand. The next step a CEO should take is to make his expectations known. He doesn’t assume that every team member knows what needs to be done. The final and most important step for a CEO to take is to foster teamwork. CEOs can achieve this by keeping communication channels open and encouraging feedback, as well as recognizing team members’ efforts and rewarding them. Setting clear goals directly from the company is important. That is why it is so important to organize regular and directed meetings by a smaller leadership core group for prompt decision making and execution of action plans in a pandemic situation mentioned in the work (Tay et al., 2021).

Another way in which CEOs can support their teams is through specialization. A CEO must be committed both to his team members and to the challenges at hand. If the CEO has doubts about the company’s mission and goals, the uncertainty will be reflected in the activities of his team members. However, if the CEO is fully committed, so will his teammates. Transformational leadership involves inspiring followers to engage in a shared vision and goals of the organization or unit. Transformational leaders tend to have more engaged and satisfied followers (Bass and Riggio, 2006). Followers or subordinates perform exceptionally well, often exceeding expectations. Private hospitals should ensure the role of leaders, in this case the CEO, is to always support their subordinates as a step to increase organizational agency in managing interactions with organizations and allow them to have more control over how, when, and where they are involved in their various personal and organizational roles (Ireland and Hitt, 1999). Related to this, a lot of previous research has said that supportive leadership is an expression of care for your followers and takes into account their unique needs (Breevaart et al., 2016). We get different things on the Personal Recognition dimension, which has the lowest value. This indicates that personal recognition does not have a major influence on leadership transformation. Bass and Riggio (2006) found that transformational factors generally have a greater correlation with ineffectiveness and satisfaction outcomes than contingent rewards. Contingent rewards usually have a greater correlation to outcomes than management by exception, particularly passive exception management. If you wait for your subordinates to make mistakes, then you correct them. This type of leadership is called “passive exception management.”

Personal recognition occurs when a leader shows that he or she values individual efforts and rewards achievement through praise and appreciation for employee efforts. Behaviors that involve performance rewards are critical to transformational leadership. Transformational leaders provide rewards, satisfaction, and satisfaction in appropriately rewarding followers when they do a good job. Transformational leaders direct their followers to “grab” their vision and internalize it so that followers become intrinsically motivated to strive for a common goal and vision. The fact that it does not include an employment contract for their performance is irrelevant as the style of leadership does not prevent them from providing specific forms of reward, in financial or other forms (Goodwin et al., 2001). In the innovative leadership variable, it can be seen that the dimension that has the greatest value is an improvement. This provides information that in leadership in private hospitals, aspects of improvement are the biggest factors needed by innovative leaders. This is of particular importance during a pandemic when flexibility and openness to organizational changes are required with a strong focus on infection prevention and control, patient and staff safety, and staff wellbeing. Then, innovative leadership is especially needed in introducing new unprecedented solutions. Quick implementation of innovative solutions during a crisis is paramount (Stoller, 2020).

In several studies, the meaning of innovation is not only creating and absorbing new value but also implementing new methods in business practices, workplace organization, or external relations, and improving and transforming managerial thinking in business models to cope with existing changes (D’Amato and Herzfeldt, 2008). Innovations can be made in products, services, production and distribution methods, organizational methods, marketing and corporate design methods. These are referred to as “product innovation,” “service innovation,” “process innovation,” “organizational innovation” and “marketing innovation.” Alotaibi et al. (2015) says that each of these methods classifies innovation based on where it takes place.

A CEO is the essence of business success. Researchers usually associate companies with their products, but it is the CEOs who come up with the products. Good CEOs develop good employees, who, in turn, develop good products for the organization. However, today’s landscape is constantly changing. Planning for the next quarter is a challenge; being committed to taking decisions that will play out in the next 5 years is even more difficult. This is where innovation in leadership is the key to turning a company into a success. Some private hospitals are faced with different problems and challenges in each industry. Improvement is needed by companies based on innovations made by the company’s CEO. In the end, many CEOs realized this. While in business, innovation leadership is critical. Executives must look beyond traditional means and explore the possibilities of incorporating innovation leadership into their methods that will lead companies to change. This is different from the Process and Routine dimension, which has the smallest value of the Innovative Leadership dimension. This indicates that processes and routines do not have a large influence. On the other hand, Calisir et al. (2013) argue that creativity occurs at the maximum level with democratic and cooperative leadership roles in organizations that have an organic structure as understood from this view. A leadership style is needed to create a work environment that encourages new ideas and manages processes well in managerial ways.

In the strategic leadership style variable, we can see that the dimension that has the greatest value is the paternalistic dimension. The impact of paternalistic leadership on individual, group, and organizational outcomes has been discussed by scholars in the fields of organizational behavior and management (Altman et al., 2013). The literature discusses the possible beneficial outcomes of paternalism for organizations: increased flexibility, decreased turnover, increased commitment, loyalty, and teamwork (Armstrong and Taylor, 2020). Paternalistic leadership is a managerial approach that involves a dominant authority figure acting as the patriarch or matriarch and treating employees and partners as if they were members of a large extended family. In exchange, the CEO expects loyalty and trust from employees, as well as compliance. In some cultures, the gender-neutral phrase “parental leadership” has replaced the words “paternalistic” or “maternalistic.” Regardless of what word is used to describe parents, employees working in such an environment are expected to understand that authority figures know what is best for the organization and trust that their CEO will always have the best interests of the employees in mind. Employees are listened to, but leaders always make the final decisions. This makes it so important for the CEO who serves in a private hospital because, basically, the final decision will be made by the CEO of the hospital.

Successful paternalistic leaders think about the big picture and consider how each decision will affect the “family.” CEOs as fathers value education and social skills and often go to great lengths to provide employees with opportunities to improve their business and interpersonal skills. The benefit of this leadership style, when carried out successfully, is that employees can work harder to complete tasks within a certain timeframe so that they can achieve, and sometimes even exceed, their goals of pleasing the CEO (parents) and bringing honor to their employees. Organization (family). What is obtained is the transactional dimension, which has the smallest dimension in the strategic leadership style variable. This is slightly different in the private hospital from the transactional approach in that the style creates a strong relationship between leadership and the ability to motivate goal achievement and increase performance through reward structures. Therefore, emphasis is placed on interpersonal communication and contingency reinforcement (Bass, 1990).

Organizations are complex organisms. Companies have different departments, values, desires, ideas, and personalities that make each one of them unique (DeCenzo et al., 2010). There is nothing wrong with these differences; in fact, they are part of what makes each company different from the next. But we need to understand that every company is different, and the individuals within a particular company are also different from each other. They are unique and have their own view of the world and business. It is the company’s overall goal to embrace that uniqueness, but their employees still identify with how the company views doing business. We can observe that companies have values, goals, and desires, and organizational identification is the degree to which companies and their employees share values, goals, desires, and goals.

In the hospital performance variable, the strongest dimension is product leadership. From these results, it can be observed that the leadership factor is very important in hospital performance so that leadership can direct the company to have better performance. This is different from the Operational Excellence dimension, which has the lowest score in hospital performance. This provides information that good operations cannot translate into good performance in private hospital companies. Leadership factors are needed that can provide direction for companies to make improvements in terms of performance. For an organization, young or old, having a breakthrough in innovation stems from an internal culture driven by a passion for innovation, a passion for excellence, and a need to keep setting new directions all the time. Product development skills are different from entrepreneurial skills. New product development requires empowering a highly talented and creative team to continuously innovate to break all records and motivate them to think outside the box. In order to be creative, you need to put together a team of people from different backgrounds who are all very creative and who can work together as a cross-functional team without fear of failing.

Transformational leaders at hospitals focus on the individual needs and development of employees, acting as mentors and motivating employees to go beyond their personal interests for the benefit of the organization. This phenomenon is a leadership style that is also characterized by an inspiring vision from the leadership that increases the pride and attachment of employees to the organization. It was also found that in this study, transformational leadership can help employees feel more connected to the hospital and improve the hospital’s performance. This is in line with other studies that found the same thing, like when transformational leaders made employees feel more connected to their work units (Epitropaki and Martin, 2005) or their organization (Walumbwa et al., 2008). In this study, it is known that organizational identification is able to provide a mediating effect on several leadership variables, as it is known that identification in organizations such as hospitals is able to provide meaning for leaders to improve organizational identification, especially during a pandemic where workers in hospitals are required to work more with patient levels, which increased drastically. Identification is able to clarify the position of health workers in hospitals to have strong associations with their organizations, no longer being individual behavior but melting into organizational behavior that has the same work spirit. Increased job demands will make the identification of organizations in hospitals stronger in carrying out their duties as health workers who serve the community during the COVID-19 crisis.

The leadership style and the implementation of policy changes for hospitals in Indonesia are the main topics discussed. Based on the concept of leadership style, there are three important dimensions of leadership that can be used during the COVID-19 crisis. First, the nature of the leader. Successful leaders are based on their personalities and why they lead an organization. Leaders must be able to influence others to improve hospital performance. The nature of the leader is able to support people who follow, have emotional intelligence, and motivation. Because strong leaders are expected to rise from adversity. The second important point, the situation, is the ability to see the existing problem situation and solve it. At this point lies the function of learning to solve problems, namely by understanding the problem. Third, connectivity, where leaders who have the ability to strategically combine knowledge, motivation and abilities form a unified effort and initiative. This connectivity includes leading to subordinates, to superiors, within the organization, as well as to parties inside and outside the organization.

COVID-19 hit the whole world, including Indonesia. The Health System became stammered and giddy. Hospitals are experiencing crises including financial crises which have a series of doctors and health workers working. The health department is having a hard time controlling and monitoring the spread of COVID-19. Health colleges with various shortcomings are trying to change the teaching system to be internet-based. Leaders of health institutions must be able to respond to crises that occur with good leadership so that the hospitals they lead can safely navigate the pandemic and continue to develop after the pandemic. The purpose of implementing this activity, first, is to understand the importance of institutional leadership during the outbreak. Second, understand the concept of leadership style that can be used in the COVID-19 pandemic. Third, use existing knowledge to develop business continuity and possible revision of the hospital’s strategic plan.

## Conclusion

Since leadership is at the heart of organizational management, it is a critical component that determines the successful operation of hospital performance. Leadership activities will demonstrate various leadership styles and their patterns. The current leadership tactics that can be applied to the healthcare setting have been impacted by a variety of ideas, instances, and models. The dynamic linkages between leadership ideals, culture, competencies, and the organizational setting should be the focus of effective leadership guidance. This dynamic must be reflected in the leader’s developmental path, which must be supported by a high level of self, team, and organizational awareness. Leadership development has clearly reached a critical juncture, with the most important task of the leader being to ensure a ready supply of replacement leaders in order to keep the company moving forward in the ever-changing healthcare environment. Hospital leaders require a diverse set of competencies to effectively respond to public health crises, particularly infectious disease epidemics. The term “competence” refers to a set of talents, values, knowledge, capacities, and capabilities possessed by people when completing a task. This study suggests for every leader to strengthen organizational identification with all forms of policy because identification will give importance to every health worker involved in improving hospital performance. The following sections highlight some of the most crucial characteristics, talents, and competences that hospital administrators must possess in order to combat COVID-19 as a public health epidemic. While no single set of attributes is required for all leadership circumstances, certain characteristics and talents are more crucial than others during times of crisis management. In order to inspire his or her staff, a hospital leader must demonstrate psychological stability and balance; he or she must also have the confidence and self-assurance required to address a crisis, manage stress, and remain calm and focused on the midst of chaos. Courage is also required to make difficult decisions about risky and perhaps divisive initiatives. They must be willing to make difficult decisions, especially when their knowledge is incomplete or limited. To deal with the outbreak effectively, healthcare leaders must be informed and aware of the current situation, have a sense of urgency, exhibit emotional intelligence, and be able to tolerate uncertainty. Because they are self-disciplined, they can keep a situation under control and deal with tasks and obstacles that seem impossible.

## Data Availability Statement

The raw data supporting the conclusions of this article will be made available by the authors, without undue reservation.

## Author Contributions

All authors listed have made a substantial, direct, and intellectual contribution to the work, and approved it for publication.

## Conflict of Interest

The authors declare that the research was conducted in the absence of any commercial or financial relationships that could be construed as a potential conflict of interest.

## Publisher’s Note

All claims expressed in this article are solely those of the authors and do not necessarily represent those of their affiliated organizations, or those of the publisher, the editors and the reviewers. Any product that may be evaluated in this article, or claim that may be made by its manufacturer, is not guaranteed or endorsed by the publisher.
